# Imitation eines Hodgkin-Lymphoms durch Frühmanifestation einer Methotrexat-assoziierten lymphoproliferativen Erkrankung

**DOI:** 10.1007/s00105-021-04804-6

**Published:** 2021-04-01

**Authors:** Maria Rosa Burg, Stefan W. Schneider

**Affiliations:** Klinik und Poliklinik für Dermatologie und Venerologie, Universitätsklinikum Hamburg-Eppendorf, Martinistr. 52, 20246 Hamburg, Deutschland

**Keywords:** Psoriasis, Pityriasis rubra pilaris, Generalisierte Lymphadenopathie, Langzeittherapie, Immunsuppressive Therapie, Psoriasis, Pityriasis rubra pilaris, Generalized lymphadenopathy, Long-term treatment, Immunosuppression therapy

## Abstract

Eine Langzeittherapie mit Methotrexat (MTX) ist als Auslöser einer MTX-assoziierten lymphoproliferativen Erkrankung (MTX-LPD) bekannt. Unter einer Kombinationstherapie mit MTX und Ustekinumab kam es bei einer 58-jährigen Patientin mit Psoriasis vulgaris und Pityriasis rubra pilaris innerhalb von 4 Monaten zu einer generalisierten Lymphadenopathie. Die Histologie deutete zunächst auf ein Hodgkin-Lymphom hin. Nur die Zusammenschau mit dem klinischen Hintergrund konnte die Diagnose einer MTX-LPD aufzeigen. Unseres Wissens ist dies der erste Fall einer MTX-LPD nach nur 4 Monaten Therapie.

## Abstract (en)

Long-term methotrexate (MTX) treatment is known to cause MTX-associated lymphoproliferative disorder (MTX-LPD). A 58-year-old woman with psoriasis vulgaris and pityriasis rubra pilaris was treated with a combination of MTX and ustekinumab for 4 months when she developed generalized lymphadenopathy. The initial histopathological analysis indicated Hodgkin’s lymphoma; however, assessing the patient’s clinical history revealed the diagnosis of MTX-LPD. To our knowledge, this is the first case of a MTX-LPD after only 4 months of treatment.

## Anamnese

**Figure 1:**
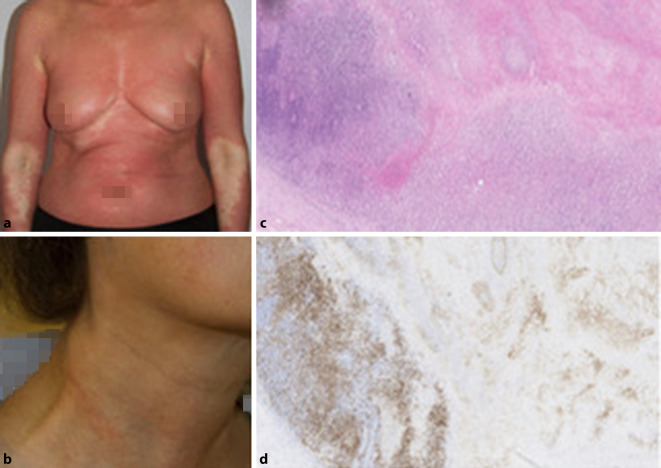

Bei einer 58-jährigen Patientin traten 07/2017 erstmalig erythematös schuppende Plaques auf. Erst nach 3 Probebiopsien konnte durch enge klinisch-histopathologische Korrelation die Diagnose einer Psoriasis vulgaris mit Pityriasis rubra pilaris gestellt werden (Abb. 1a).

Da sich eine Therapie mit Retinoiden bei ausgeprägter Xerosis cutis und massivem Haarausfall als relativ kontraindiziert erwies, initiierten wir nach Erstvorstellung in domo 08/2018 eine Behandlung mit Methotrexat (MTX) 10 mg 1‑mal wöchentlich und dem Interleukin-12/23-Inhibitor Ustekinumab 45 mg 1‑mal alle 3 Monate.

Vier Monate nach Therapiebeginn mit MTX kam es zu einer neu aufgetretenen, schmerzhaften, generalisierten Lymphadenopathie, Müdigkeit und Schwäche bei sonst fehlender B‑Symptomatik.

## Befund

Klinisch zeigten sich lumbal und okzipital in Abheilung befindliche, schuppende, bräunliche, scharf begrenzte Plaques. Die Lymphknoten zervikal, supraklavikulär sowie inguinal waren bereits makroskopisch sichtbar, nicht druckschmerzhaft und zeigten sich auch sonographisch deutlich vergrößert (Abb. 1b).

Eine diagnostische Lymphknotenexstirpation zervikal rechts zeigte einen partiell stark strukturalterierten Lymphknoten mit ausgeprägter Nekrose und Epstein-Barr-Virus(EBV)-positiver Blastenproliferation im vitalen Randbereich, pleomorphen Blasten mit prominenten Nukleoli, positiv für CD30, CD15 und PAX5 bei Negativität gegenüber CD20, CD3 und LCA/CD45, mit stellenweiser Eosinophilie im Begleitinfiltrat. Eine klonale Genumlagerung des Immunglobulin-Schwerketten-Genlocus Immunglobulin H (IgH) war nicht nachweisbar (Abb. 1c, d).

## Diagnose

Der Befund wurde zunächst von unseren Hämatoonkologen als Lymphknotenbefall durch ein klassisches Hodgkin-Lymphom mit ausgeprägten Nekrosen vom gemischtzelligen Typ gewertet. Auch eine referenzhistopathologische Begutachtung bestätigte ein klassisches Hodgkin-Lymphom mit ungewöhnlicher Präsentation im histologischen Bild, ausgedehnten Nekrosen und teilweise fibrohistiozytischer Proliferation.

Erst nach erneutem Austausch über den klinischen Hintergrund konnte man die Veränderungen als Immuninsuffizienz auf dem Boden einer PTLD(„post-transplant lymphoproliferative disorder“)-ähnlichen Erkrankung nach immunkompromittierender Therapie mit MTX einordnen, die sich morphologisch und immunhistochemisch kaum bzw. nicht vom Hodgkin-Lymphom unterscheiden lässt. Für eine PTLD sprachen auch die ausgedehnten Nekrosen des Lymphknotens vor dem Hintergrund des klinischen Verlaufs.

## Therapie und Verlauf

Bei initialem Verdacht auf eine durch MTX-induzierte Lymphadenopathie wurden die Systemtherapeutika MTX sowie Ustekinumab abgesetzt.

Die Vorbereitung einer bei Verdacht auf Hodgkin-Lymphom geplanten Chemotherapie nach eskaliertem BEACOPP-Protokoll wurde erst nach erneutem klinisch-histopathologischem Austausch abgebrochen.

Nach Absetzen von MTX kam es innerhalb von 6 Monaten zur vollständigen Remission der Lymphknotenschwellungen, sodass wir zur weiteren Stabilisierung des Hautbefundes die Therapie mit Ustekinumab mit kompletter Abheilung aller Hautläsionen und wieder normaler Haardichte bei guter Verträglichkeit fortführten.

## Diskussion

MTX-assoziierte lymphoproliferative Störungen (MTX-LPD) stellen eine iatrogene Erkrankung dar und werden nach der WHO(Weltgesundheitsorganisation)-Klassifikation 2017 der Gruppe der anderen iatrogenen Immundefizienz-assoziierten lymphoproliferativen Störungen zugeordnet, die sich durchaus bei 13,3 % der Fälle histologisch als klassischer Hodgkin-Lymphom-Typ darstellen [6].

MTX-LPD im Sinne einer lymphoiden Proliferation oder Lymphom nach Immunsuppression mit MTX wurden meist bei Patienten mit rheumatoider Arthritis, aber auch vereinzelt bei Psoriasis vulgaris beschrieben [3, 7]. Jedoch treten diese meist erst nach langjähriger Therapie (durchschnittlich 54 Monate) auf [2].

Dies ist unseres Wissens bisher der erste in der Literatur beschriebene Fall einer MTX-LPD, der bereits nach 4 Monaten Therapie mit MTX aufgetreten ist.

Alleine durch Absetzen von MTX können MTX-LPD – wie bei der von uns beschriebenen Patientin – reversibel sein [5]. Bei weiterer Progredienz ist jedoch auch ein Übergang in ein High-grade-Lymphom (EBV-assoziiertes High-grade-Lymphom) oder ein definitives Hodgkin-Lymphom möglich [8].

Als prognostische Marker für den klinischen Verlauf wurden der Nachweis von EBV, Monoklonalität von IgH sowie der histologische Subtyp beschrieben [4].

MTX könnte möglicherweise aufgrund seiner immunsuppressiven Eigenschaften und der Reaktivierung von EBV in latent infizierten B‑Zellen EBV-positive Lymphome auslösen [1].

## Fazit für die Praxis

- Eine immunsuppressive Therapie, insbesondere mit Methotrexat (MTX), sollte stets als möglicher Auslöser einer Lymphknotenschwellung im Sinne einer lymphoproliferativen Erkrankung in Erwägung gezogen werden.
- Dieser Fall zeigt eindrücklich, wie wichtig Beständigkeit bei der Diagnosefindung und eine interdisziplinäre Zusammenarbeit mit steter klinisch-histopathologischer Korrelation sind, um fälschlicherweise durchgeführte und möglicherweise mit starken Nebenwirkungen verbundene Therapien vermeiden zu können.
